# Hoverfly locomotor activity is resilient to external influence and intrinsic factors

**DOI:** 10.1007/s00359-015-1051-2

**Published:** 2015-11-26

**Authors:** Malin Thyselius, Karin Nordström

**Affiliations:** Department of Neuroscience, Uppsala University, Box 593, 751 24 Uppsala, Sweden; Anatomy and Histology, Centre for Neuroscience, Flinders University, GPO Box 2100, Adelaide, SA 5001 Australia

**Keywords:** Diet, Age, Sexual dimorphism, Circadian rhythm, Starvation

## Abstract

Hoverflies are found across the globe, with approximately 6000 species described worldwide. Many hoverflies are being used in agriculture and some are emerging as model species for laboratory experiments. As such it is valuable to know more about their activity. Like many other dipteran flies, *Eristalis* hoverflies have been suggested to be strongly diurnal, but this is based on qualitative visualization by human observers. To quantify how hoverfly activity depends on internal and external factors, we here utilize a locomotor activity monitoring system. We show that *Eristalis* hoverflies are active during the entire light period when exposed to a 12 h light:12 h dark cycle, with a lower activity if exposed to light during the night. We show that the hoverflies’ locomotor activity is stable over their lifetime and that it does not depend on the diet provided. Surprisingly, we find no difference in activity between males and females, but the activity is significantly affected by the sex of an accompanying conspecific. Finally, we show that female hoverflies are more resilient to starvation than males. In summary, *Eristalis* hoverflies are resilient to a range of internal and external factors, supporting their use in long-term laboratory experiments.

**Electronic supplementary material:**

The online version of this article (doi:10.1007/s00359-015-1051-2) contains supplementary material, which is available to authorized users.

## Introduction

Hoverflies are named after their ability to hover near stationary for prolonged periods of time. They are found across the globe, with ca. 6000 species described worldwide. The hoverfly *Episyrphus balteatus* is important in agriculture where it is used for biological control of aphids (Tenhumberg and Poehling 1995) and for pollination (Jauker et al. 2009). Species of the larger *Eristalis* genera are also important for pollination (Gladis 1997), and are additionally emerging as important model organisms for investigating widely different scientific questions, such as the neuronal basis of visual processing (De Haan et al. 2012), how captivity affects the gene pool (Francuski et al. 2014), and flight kinematics (Walker et al. 2012). It is therefore valuable to know more about hoverfly locomotor activity levels.

Most animals show a strong preference for diurnal, nocturnal or crepuscular locomotor activity (see, e.g., Mistlberger and Skene 2004 and Lewis and Taylor 1965 for reviews on mammalian and insect circadian rhythms). Many insects, including assassin bugs and the dipteran model *Drosophila*, seem to be mainly crepuscular, with peak activity at sunrise and sunset (Long et al. 2014; Lazzari 1992). Hoverflies, like many other dipteran flies, such as the blowflies *Phormia* (Green 1964) and *Calliphora* (Cymborowski et al. 1994), and robber flies (Lee et al. 2014), have been described as diurnal (Gilbert 1985; Ottenheim 2000). However, these results were based on the hoverflies being visualized and caught by human observers, such as by hand-netting hoverflies in 2 h intervals (Ottenheim 2000). It is thus possible that the results were distorted by the sensitivity and precision of the human sensory–motor systems.

Many factors affect an animal’s incentive to move, and subsequently there are substantial differences in locomotor activity during the day as well as between the seasons. Furthermore, locomotion is used in widely different behaviors, such as migration (Sane et al. 2010), for finding food (Lighton and Duncan 2002; Lewis et al. 2008; Vinauger et al. 2011) or shelter (Whitaker and Shine 2003), locating conspecifics (Smith et al. 1994) or avoiding predators (Weihmann et al. 2010; Bulbert et al. 2015). Locomotion and physical activity patterns are subsequently affected by both external factors, such as the presence or absence of conspecifics (Hoffmann 1990) or predators (van der Bijl et al. 2015), as well as by internal factors, such as the age (Rakshit et al. 2013), hormonal status (Pflüger and Duch 2011) and sex (Minoli and Lazzari 2006) of the animal. Thus, in many animals, locomotor activity is sexually dimorphic. Housefly males, for example, are significantly more active than females under a range of different temperatures and housing densities (Bahrndorff et al. 2012; Schou et al. 2013). Indeed, increasing the number of males in a group of houseflies increases the activity of the entire group (Bahrndorff et al. 2012). Houseflies are sexually dimorphic in walking behavior (Bahrndorff et al. 2012; Schou et al. 2013) as well as in free flight behavior (Wehrhahn 1979). Many hoverflies also show strong sexually dimorphic flight behavior, where males set up territories that they vigorously defend against intruding conspecifics (Collett and Land 1975; Fitzpatrick and Wellington 1982). An intruding male is chased away from the territory, whereas females are pursued for courtship and mating (Fitzpatrick 1981). The sexually dimorphic behavior is accompanied by sexually dimorphic eye design (Collett and Land 1975) and neurophysiology (Nordström et al. 2008).

In many animals locomotor activity changes with age. A general decrease in locomotor activity has been observed in widely different species, such as killifish (*Nothobranchius korthausae*, Lucas-Sanchez et al. 2011), humans (Troiano et al. 2008) and rats (Peng and Kang 1984). However, in the fruit fly *Drosophila,* the influence of age differs between strains. In one study comparing five different wild-type strains, three of these showed a constant activity throughout their life (Fernandez et al. 1999), whereas activity decreased with age in the other two. When starved, many animals, including rats and mice (Patton and Mistlberger 2013), goldfish (Vera et al. 2007), medaka (Weber and Spieler 1987), insects and primates (for review, see Mistlberger 1994), display an anticipatory increase in activity before expected food delivery. If food is not provided when expected, the activity increases substantially with the aim of locating food sources before internal energy supplies run too low. The increased activity is a true food searching response, since the activity drops immediately when food is provided (Green 1964; Stevenson and Rixon 1957). Furthermore, in both vertebrates and invertebrates, the activity levels are affected by the diet provided (Croy and Hughes 1991; Catterson et al. 2010). This is important since *Eristalis* hoverflies have been reared on very different diets in laboratory conditions, such as either honey and pollen (De Haan et al. 2013), sugar (Horridge et al. 1975) or honey (Wacht et al. 2000), and it is unclear what effect the diet may have had. In the wild, hoverflies eat pollen and nectar from the same type of flowers as bees typically feed on (Golding and Edmunds 2000).

In *Drosophila*, locomotor activity can be rapidly quantified using an activity monitor that is equipped with infrared beams that break each time the fly walks through a glass tube (e.g., Pfeiffenberger et al. 2010; Tataroglu and Emery 2014). Recently, a larger version of this activity monitor has become available, allowing quantification of the activity of larger insects, such as houseflies (Bahrndorff et al. 2012; Schou et al. 2013). This type of activity monitor does not distinguish between different types of locomotion, such as grooming, walking, running or flying, as it simply quantifies the number of times the animal passes a certain point in space. However, the activity monitor allows for rapid quantification of the general level of an animal’s activity, which makes it valuable for investigating the effect of many different factors.

To quantify the effect of different internal and external factors on hoverfly activity levels, we therefore here utilize the locomotor activity monitoring system (Bahrndorff et al. 2012; Schou et al. 2013; Pfeiffenberger et al. 2010). We confirm that *Eristalis* hoverflies are diurnal, with a near constant activity during the 12 h the light is on during a 12 h light:12 h dark (LD) cycle, with close to no activity during the dark hours of the night. If the light is turned on during the night, the activity increases slightly, but not to day-time levels. We further show that hoverfly locomotor activity is remarkably stable over the lifetime of the animals, and also resilient to the diet provided. Somewhat surprisingly, we find that locomotor activity is sexually isomorphic when the animals are solitary, but that the activity is significantly affected by the sex of an accompanying conspecific. Finally, female hoverflies are more resilient to starvation than males, supporting the observation that only females appear to overwinter in temperate climates (Dennys 1927; Kendall and Stradling 1972). We conclude that *Eristalis* hoverflies are resilient to a range of external and internal factors, making them suitable as laboratory models.

## Materials and methods

### Fly rearing

*Eristalis tenax* (Linnaeus) larvae were collected from cow dung or purchased as pupae (Bioflytech, Alicante, Spain) and reared till hatching in a double-layered net (~2.5 m^3^) suspended from the ceiling, subjected to a 12 h light:12 h dark (LD) cycle, at 21.5° ± 2.5 °C. Newly hatched adults had access to a pollen–sugar mix and water ad libitum. Within three days of hatching, male and female adults were transferred to a fridge in 24 h darkness (DD), at 4 °C. For the virgin experiments, the two sexes were kept separate immediately from hatching. Twice a week, the hoverflies were taken out to room temperature (20–22 °C), room light, and fed a pollen–honey–water mix ad libitum for approximately 5 h, before being returned to the fridge.

### Activity rhythms

We used a Locomotor Activity Monitoring system (LAM25, TriKinetics Inc, Waltham, MA, USA) with 25 mm diameter × 125 mm long Pyrex glass tubes (PGT25 × 125, TriKinetics Inc) positioned horizontally. Each tube contained one hoverfly, unless otherwise stated. All recordings were performed in 12:12 LD, using daylight fluorescent lamps (58 W/865, Helsingborg, Nova Group AB, Sweden), unless otherwise stated. The temperature was 21.5° ± 2.5 °C, similar to that of a temperate summer day when hoverflies tend to be active (Gilbert 1985; Ottenheim 2000). Each experiment was started in the middle of the 12 h light cycle and lasted approximately 54 h, unless otherwise stated.

In all experiments, activity was measured as beam breaks of a photocell in the middle of the Pyrex glass tube, at a frequency of min^−1^. When two flies were placed in the tube, we divided the total activity by 2, to get a comparative activity frequency of min^−1^ fly^−1^. During our pilot experiments, we discovered that sometimes a hoverfly would sit for a prolonged period of time in the middle of the tube, continuously breaking the beam, and thus generating an unrealistic number of activity counts. To remove these potential false positives from the data, we first identified all continuous measurements of over 10 counts/min and then replaced the first one of these with 1 count/min, followed by 0 counts/min.

To confirm that the LAMS provides a fair representation of the general level of activity, we filmed 8 tubes (Supp Movie 1), and analyzed the hoverfly activity from the films manually (Supp Fig. 1). This analysis (Supp Fig. 1) shows that the LAMS captures the general activity of the animals.

### Diets

In all experiments, both ends of the tubes were sealed with a moist organic cotton ball (à la eco AB, Bromölla, Sweden). In most experiments, these were dipped in a solution of honey, pollen, and water. For longer experiments, the cotton balls were changed and rehydrated every second day, during the dark phase of the experiment, guided by a red light source (with a peak at 655 nm).

To investigate the effect of diet, we used hoverflies that were 2.5–3.5 months old. The hoverflies had access to water from the moist cotton balls that sealed the ends of the tube. Besides the water, the flies had access to one of six different diets or a starvation control (water only). There were four single diets: sugar, pollen, nectar and honey, and two combination diets: sugar and pollen, or honey and pollen.

To investigate the effect of starvation, we performed an experiment that lasted for 7 full days. The starved flies only had access to water, whereas the control flies had access to water, honey and pollen. To quantify survival, we counted the number of flies that were alive at the end of the light period each day.

### Analysis and statistics

Matlab (Mathworks, Natick, MA, USA) and Graphpad Prism 6 (GraphPad Software Inc., La Jolla, CA, USA) were used for statistical analysis. Data from different experiments were aligned to the start of the light phase. We quantified the mean activity during 4 h in the middle of the light period of day 2, except for the starvation experiment where we quantified the mean activity during 4 h in the middle of the light period for 7 consecutive days. We used the second day for statistical analysis, since several previous papers (van der Voet et al. 2015; Freeman et al. 2010; Gershman et al. 2014; Jepson et al. 2011; McCarthy et al. 2015) let the flies acclimatize for 12–24 h before quantification. Importantly, our conclusions do not depend on the size of the analysis window (Supp Fig. 2).

For analysis of significance, we first performed a D’Agostino and Pearson omnibus normality test. Where data were normally distributed we did a paired *t* test, one-way ANOVA combined with Sidak’s multiple comparisons test, two-way ANOVA with Tukey’s post hoc test, or three-way ANOVA, depending on data set. When the data could not be shown to be normally distributed, a Mann–Whitney test or a Kruskal–Wallis test with Dunn’s post hoc test was used. The appropriate test is given in the text or in the relevant figure legend. In all cases, significance was allocated to four levels, with *P* values below 0.05, 0.01, 0.001 and 0.0001 indicated with 1, 2, 3 or 4 stars (*), respectively.

Most of the data are displayed with box plots, where the central mark shows the median and the edges of the box the 25th to 75th percentiles of the data. The whiskers extend from the 5th to 95th percentiles of the data, and any outliers are indicated with crosses (×). When numbers are cited in the text, we give the mean ± SD, unless otherwise stated. In all cases, *N* = number of experiments and *n* = number of hoverflies, with the data averaged across the number of animals (*n*). Except for the starvation experiment, we only show data for hoverflies that stayed alive for the full duration of the experiment.

## Results

### *Eristalis tenax* hoverflies show a strong diurnal rhythm

**Fig. 1:**
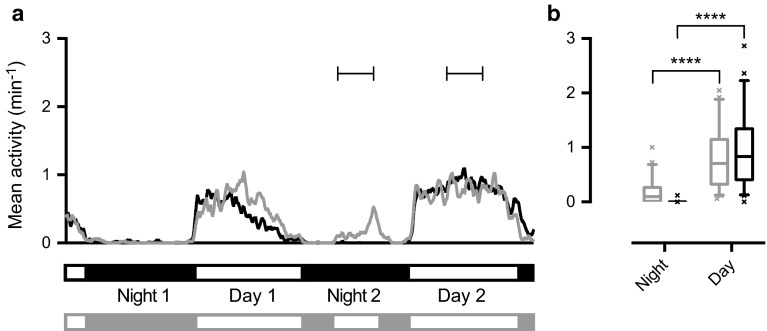
*Eristalis tenax* hoverflies are active during the day and inactive at night. **a** The *black data* show the locomotor activity of 43 *Eristalis tenax*, measured as beam breaks sampled every minute in a LAMS. In this panel, the mean data are low pass filtered purely for illustrative purposes (using a first order Butterworth low pass filter with a cutoff frequency of 0.04). The *black bar* under the data shows the 12:12 LD cycle. The *gray data* show the activity of 45 *Eristalis tenax* where the light was turned on for 5 h during the second night. The *gray bar *under the data shows the light-dark cycle. All hoverflies were 1–2 months old. *Brackets* show the 4 h used for statistical analyses. **b** The *data* show the mean activity during 4 h in the middle of night 2 and day 2 (extracted from the data in panel **a**). Significance was tested with a two-way ANOVA followed by a Tukey’s post hoc test (*P* < 0.0001)

To quantify the diurnal activity of *Eristalis tenax,* we here used a Locomotor Activity Monitoring system (LAMS) to measure their general locomotor activity. For this purpose, we placed 1- to 2-month-old hoverflies in individual Pyrex tubes. The activity was measured for 54 h under a 12 h light:12 h dark (LD) cycle, with the experiment started in the middle of a light cycle. *E. tenax* hoverflies displayed a robust diurnal activity pattern with continuous activity during the 12 h of light, and no activity during the 12 h of darkness (black data, Fig. 1a, *n* = 43, *N* = 1).

Since the hoverflies appeared to be active for the entire 12 h light period (black data, Fig. 1a), we pooled the activity over several hours for statistical analyses. The black data in Fig. 1b show a comparison of the activity averaged over 4 h in the middle of the second night and day (analysis windows indicated with brackets in Fig. 1a). Note that the conclusions do not depend on the size of the analysis window (Supp Fig. 2). Here, and throughout the paper, the data are displayed using box plots where the central mark indicates the median, the edges of the box the 25th to 75th percentile, and the whiskers the 5th to 95th percentiles.

To investigate whether the activity pattern (black data, Fig. 1) is driven by an internal circadian rhythm or by the external light, we turned the light on for 5 h during the second night (gray data, Fig. 1a, *n* = 45, *N* = 1). This analysis showed that even if the light during the night increased hoverfly activity (gray data, Fig. 1a), this increase was not significantly different to the night when the light was off (black data, Fig. 1b). Furthermore, the night activity level was much lower than the activity during the day (*P* < 0.0001, two-way ANOVA, gray data, Fig. 1b). Importantly, turning the light on for 4 h during the night did not affect the activity level during the following day (compare black and gray data, Fig. 1).

### Hoverflies show a stable activity throughout life

**Fig. 2:**
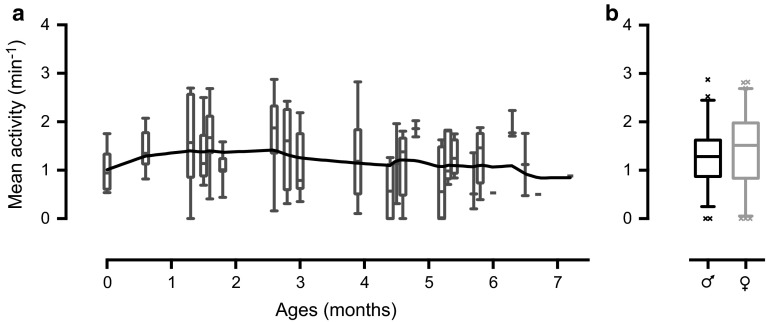
Hoverfly activity remains robust in aging hoverflies. **a** The *box plots* show the mean hoverfly activity as a function of age. The data show the activity for male and female hoverflies, with the black line showing a fitted lowess curve. No difference was found between the different ages (Kruskal–Wallis test with Dunn’s post hoc test). Starting from the *box plot* on the *left*, *n* = 6, 9, 7, 8, 10, 10, 15, 4, 8, 9, 6, 2, 6, 2, 4, 7, 4, 3, 5, 1, 3, 2, 1 and 1; *N* = 16. **b** The mean activity of male (*black*, *n* = 58, *N* = 14) and female (*gray*, *n* = 75, *N* = 15) hoverflies. The activity was averaged during 4 h in the middle of day 2. No significant difference was found (Mann–Whitney test)

To investigate how locomotor activity is affected by age, we quantified the activity of hoverflies that increased in age from those that had just hatched to the oldest ones we were able to keep alive in the lab. The data show that although the youngest (0 months) and oldest (7 months) hoverflies had a slightly lower activity, there was no significant effect of age (Kruskal–Wallis test with Dunn’s post hoc test). Rather, hoverflies of all ages had a stable activity level of ca. 1.4 ± 0.8 min^−1^ (Fig. 2a). Furthermore, we noted that the hoverflies appeared to be healthy up to a high age, since females as old as 5.4–5.8 months laid fertile eggs.

Importantly, in many animals, locomotor activity not only depends on the age of the animal, but also on its sex. We therefore additionally compared the activity level between male and female hoverflies. This analysis showed no significant difference between the walking activity of the two sexes (males 1.3 ± 0.6 min^−1^, *n* = 58, *N* = 14; females 1.4 ± 0.8 min^−1^, *n* = 75, *N* = 15, Fig. 2b, Mann–Whitney test).

### The effect of company

**Fig. 3:**
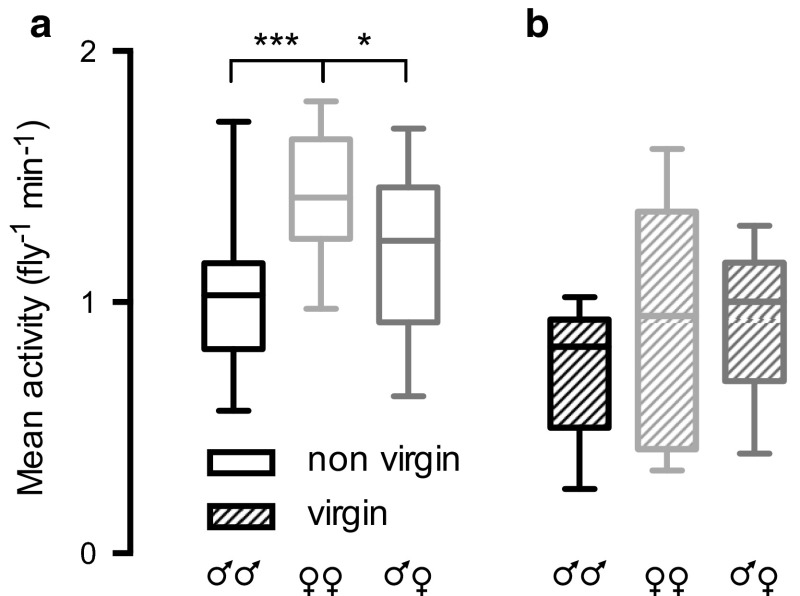
Locomotor activity is affected by company. **a** The *data* show the mean activity per fly when there were two hoverflies in each tube. Each tube housed either two males (♂♂, 16 tubes with 32 hoverflies, *N* = 3), two females (♀♀, 15 tubes with 30 hoverflies, *N* = 3) or one male and one female (♂♀, 18 tubes with 36 hoverflies, *N* = 3), aged 1–2 months. Significance was tested with a one-way ANOVA with Sidak’s post hoc test, with *1 star* (*) for *P* < 0.05, and* 3 stars* (***) for *P* < 0.001. **b** The *data* show the mean activity per fly when there were two virgin hoverflies in each tube. Each tube housed either two males (♂♂, 6 tubes with 12 virgin hoverflies, *N* = 1), two females (♀♀, 5 tubes with 10 virgin hoverflies, *N* = 1) or one male and one female (♂♀, 5 tubes with 10 virgin hoverflies, *N* = 1), aged 0.4–2 months. Significance was tested with a one-way ANOVA with Sidak’s post hoc test (ns)

It is striking that we saw no difference between male and female activity (Fig. 2b). Can sexually dimorphic behavior be induced by the presence of a conspecific? To investigate this hypothesis we placed two hoverflies in each tube and measured the resulting locomotor activity. We found that the activity per male fly (1.0 ± 0.3 min^−1^, Fig. 3a) was slightly lower than when male hoverflies were placed in the tubes individually (1.3 ± 0.6 min^−1^, Fig. 2b), but that the female activity was unchanged (1.4 ± 0.3 min^−1^, Fig. 3a, compared with 1.4 ± 0.8 min^−1^ for the individuals, Fig. 2b).

Interestingly, with two hoverflies in each tube, the sex of the tube mate influenced locomotor activity (Fig. 3a). When two females were paired together they showed a 22 % higher activity per fly than when one male and one female were paired together (Fig. 3a, *P* < 0.01, one-way ANOVA with Sidak’s post hoc test). Two females paired together showed a 38 % higher activity per fly than two males in the same tube (Fig. 3a, *P* < 0.001, one-way ANOVA with Sidak’s post hoc test).

We next investigated if the activity is affected by the mating status of the tube mate by repeating the experiment with virgin animals (Fig. 3b). The activity of the virgin animals tended to be lower than the activity of the non-virgins (but not significantly, Kruskal–Wallis test with Dunn’s post hoc test). We found no significant difference between the three combinations of sexes (male–male, male–female or female–female) when they were virgins at the start of the experiment (Fig. 3b, Kruskal–Wallis test). In summary, the data in Fig. 3 show that *Eristalis* walking behavior is affected by the sex of the conspecific in its vicinity, when the conspecific comes from a mixed-sex population.

### Diet has a small effect on activity but influences male mortality

**Fig. 4:**
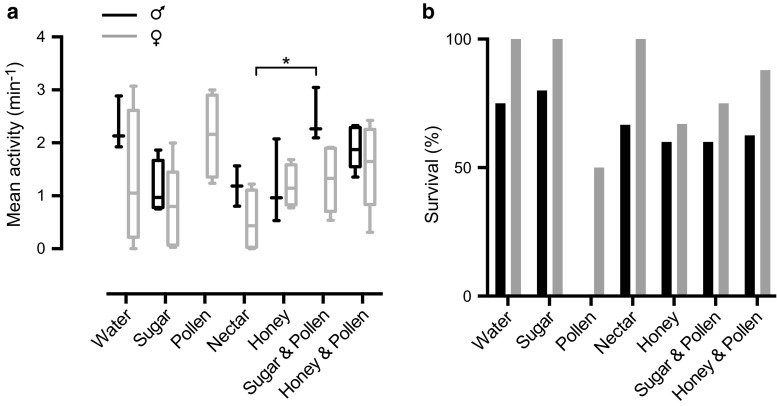
Sexually dimorphic dependence on diet. **a** The *data* show the activity as a function of diet for 2.5–3.5 months old hoverflies. We only show the activity for the hoverflies that survived the whole experiment. Starting from the *box plot* on the *left*, *n* = 3, 4, 4, 5, 4, 2, 4, 3, 4, 3, 6, 5 and 7, *N* = 6. Female activity is shown in *gray* and male activity in *black*. The *star* shows a significant difference (*P* < 0.05, two-way ANOVA with Tukey’s post hoc test). **b** The *data* show the percentage of hoverflies that survived until the end of the 2nd day, with female survival in *gray* and male survival in *black*. The data include the hoverflies in panel (**a**), but also those that died during the experiment, thus *n* = 4, 4, 5, 5, 6, 8, 3, 4, 5, 6, 5, 8, 8 and 8, *N* = 6

Lab hoverflies in the literature have been fed a variety of diets (e.g., De Haan et al. 2013; Horridge et al. 1975; Wacht et al. 2000). We here investigate the effect of diet in the LAMS. The hoverflies had access to either water alone, water and a single food source (sugar, pollen, nectar or honey, Fig. 4a), or water and a combined food source (sugar and pollen, or honey and pollen, Fig. 4a). Since Gilbert (1981) observed a sexual dimorphism in the dietary preference, we quantified the data for the two sexes separately. We found that the type of diet had a very small effect on the activity (Fig. 4a, two-way ANOVA with Tukey’s post hoc test).

Strikingly, even though we found virtually no sexually dimorphic locomotor activity dependence on diet (Fig. 4a), the female hoverflies (gray data, Fig. 4b) showed much higher survival than the males (black data, Fig. 4b). Male survival rate showed a strong dependence on diet (black data, Fig. 4b). Indeed, not a single male survived the 54 h on a diet of only pollen (black data, Fig. 4b), but they fared much better under the other dietary regimes, including water only.

### Hoverfly activity is not influenced by starvation

**Fig. 5:**
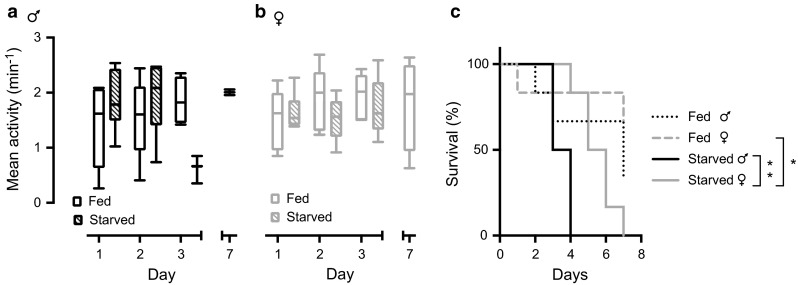
Females are more resilient to starvation. **a** The data show how male hoverfly activity is affected by starvation (*dashed boxes*, *n* = 6 for day 1 and day 2, *n* = 3 for day 3, *N* = 1). The clear *box plots* show the control data where the hoverflies had free access to pollen and honey (*n* = 6 for day 1, *n* = 5 for day 2, *n* = 4 for day 3, and *n* = 2 for day 7, *N* = 1). **b** The same data but for female hoverflies (starved, *n* = 6 for day 1–3; fed, *n* = 5 for day 1–3, and *n* = 4 for day 7). We found no effect of treatment, sex or starvation duration on activity (three-way ANOVA). **c** Survival curve for the data from panels **a** and **b** (*n* = 6, *N* = 1). There is a difference between the starved conditions (*P* = 0.003) and the starved females and their controls (*P* = 0.02; Log-rank test with Bonferroni correction for multiple comparisons)

Many animals display an anticipatory increase in activity when starved (e.g., Patton and Mistlberger 2013; Vera et al. 2007; Weber and Spieler 1987; Mistlberger 1994). To investigate whether hoverflies display such an anticipatory increase in activity during prolonged starvation, male and female hoverflies were starved for 7 days and their activity compared to control groups that were fed the standard diet of honey and pollen. Surprisingly, we found no increased locomotor activity in either males (Fig. 5a) or females (Fig. 5b, three-way ANOVA revealed no effect of treatment, sex or day). Indeed, in female hoverflies, the activity of the starving animals was remarkably similar to the locomotor activity of the fed hoverflies, even after several days (Fig. 5b).

The activity of the starving male hoverflies reduced sharply during the third day (Fig. 5a), associated with them dying (black data, Fig. 5c). Indeed, all the starving males died within 4 days (black data, Fig. 5c), whereas the last starving female survived until the 7th day (gray data, Fig. 5c). This difference in survival was significant (*P* = 0.003, log-rank test with Bonferroni correction for multiple comparisons).

Most of the female control flies survived the 7 days in the LAMS (gray dashed data, Fig. 5c), whereas two of the male controls died during the first few days (black dashed data, Fig. 5c). Subsequently, there was a significant difference between the starved females and their controls (Fig. 5c, *P* = 0.02, log-rank test with Bonferroni correction for multiple comparisons), but not between the starved males and their controls. We thus conclude that whereas the average locomotor activity is not affected by starvation (Fig. 5a, b), females survive starvation and enclosure (gray data, Fig. 5c) much better than males do (black data, Fig. 5c).

## Discussion

We have here showed that *Eristalis tenax* have a strong diurnal rhythm, being mainly active during the light period of the day, thus supporting Ottenheim’s (2000) field observations. The activity seemed to be predominantly driven by an internal circadian rhythm rather than by the external light (Fig. 1). We further showed that *Eristalis* hoverflies are remarkably resilient to external as well as internal factors, since neither age (Fig. 2a), sex (Fig. 2b), diet (Fig. 4a), nor starvation (Fig. 5a, b) seemed to have any strong effect on their locomotor activity. *Eristalis* hoverflies are thus robust, and survive laboratory conditions very well, supporting their use in long-term experiments.

The LAMS provides a method for rapidly quantifying the influence of a range of internal and external factors on general activity levels. Importantly, however, the LAMS does not separate between different types of behavior, such as mating, flying, walking and grooming. Instead, it provides a digital count of every time a beam is broken by a fly passing an infrared beam. Naturally, this type of analysis will therefore never give the same level of detail as, e.g., filming the animals and subsequently using sophisticated software to cluster the activities into different behavioral patterns (Braun et al. 2010; Geurten et al. 2010; Zimmerman et al. 2008). However, our own comparison of a manual analysis of a film with 8 hoverflies, with the data scored by the LAMS system itself (Supp Fig. 1 and Supp Movie), suggests that the LAMS scoring gives a fair representation of the general level of activity in the tubes. For rapid quantification of general levels of activity, the LAMS is thus a reliable technique.

### The influence of sex on activity

In many animals, there is a pronounced difference in activity levels between males and females, often associated with sexually dimorphic roles in, e.g., territorial defense, maintenance of social structures, or nurturing of the young. For example, in hoverflies, the observed territoriality is strongly sexually dimorphic (Fitzpatrick 1981). Despite this, in our experiments, we found no significant difference between male and female locomotor activity (Fig. 2b). Similarly, Bahrndorff et al. (2012) found no difference between male and female houseflies in a similar setup, even if they are sexually dimorphic in free flight (Wehrhahn 1979). Therefore, it is possible that the sexually dimorphism observed in free flight behaviors (Fitzpatrick and Wellington 1982, 1983; Fitzpatrick 1981) do not carry through into the walking behaviors recorded in our setup (Fig. 2b).

Even if the activity of single males and females was sexually isomorphic (Fig. 2b), we found that the sex of a conspecific in the vicinity affected locomotor activity (Fig. 3a). It is thus possible that visual and/or chemical input from the conspecific reveals its sex, affecting the activity pattern of nearby conspecifics. We found that two females in a tube were only slightly more active than single females (2 %, compare Figs. 2b, 3a). In houseflies, however, single females were more active than several females together (Bahrndorff et al. 2012). Bahrndorff et al. (2012) also noted that the more *Musca* males were present in a group, the higher the activity of each fly. However, in mixed-sex groups, the highest activity was found when there was only one male and one female (Schou et al. 2013). Furthermore, in single-sex *Musca* groups, three males in a tube were more active than a single male, but a single female was more active than three females kept together (Bahrndorff et al. 2012).

This is somewhat contradictory to our findings, where two females paired together were more active than one male and one female, or two males paired together (Fig. 3a). Importantly, however, houseflies and hoverflies differ at the neuronal level (Buschbeck and Strausfeld 1997) and in their free flight behavior (Collett and Land 1978; Land and Collett 1974). It might be possible that it is easier for housefly males to become aggressive in confined spaces and that hoverfly males need a larger space to increase their aggressive activity towards another male. Indeed, male hoverflies defend much larger territories (Fitzpatrick 1981) than houseflies (Zeil 1986) do.

### Age, diet and starvation

We found that the activity levels of hoverflies remained remarkably robust across a large range of ages (Fig. 2a), supporting their use in long-term experiments. Our observations are thus similar to work on another dipteran, the model fly *Drosophila,* where three of five strains showed a stable activity through their 3 months of life (Fernandez et al. 1999).

When blowflies (Green 1964) and *Drosophila* (Lee and Park 2004) are starved, they increase their activity in search of food. In our experiments, we saw no change in activity levels, not even after 7 days of starvation. This suggests that starvation did not induce the strong food-seeking behavior seen in many other animals (Green 1964; Stevenson and Rixon 1957). However, we did see a sudden drop in male activity just before death (Fig. 5a), just as in the flies *Drosophila* and *Phormia* (Green 1964; Lee and Park 2004).

Gilbert (1981) noted that hoverflies of both sexes feed on nectar and pollen, but that males eat less pollen than females, which could explain why males did not survive on a pollen only diet (black data, Fig. 4b). Note, though, that in our experiments, both males and females survived on a nectar only diet (Fig. 4b). Overall, we found that females survived starvation much better than males (Fig. 5c). Since fat storage and body size are often sexually dimorphic, sex affects the effect of starvation. Indeed, female *Calliphora* are larger and have a higher proportion of body fat than males (Ujvari et al. 2009). Female *Drosophila* (Hillesheim and Stearns 1991), *Episyrphus* (Putra and Yasuda 2006), and lab reared *Eristalis arbustorum* (Ottenheim and Holloway 1995) are also larger than males of the same species. Whereas we found no significant difference between male and female *Eristalis* weights, all the largest individuals were females (Supp Fig. 3).

In summary, we conclude that *Eristalis* hoverflies are robust and resilient against a range of internal and external factors, supporting their use in long-term laboratory experiments.

## Electronic supplementary material

Supplementary material 1 (JPEG 2331 kb)

Supp Fig. 1. Manual analysis of hoverfly activity. We filmed 8 hoverflies (Supp Movie 1) and the activity was simultaneously recorded using the LAMS. The top table shows the data quantified by the LAMS, with the activity color coded into three levels. The lower table shows the same data after manual analysis of the film (Supp Movie 1). The letters denominate the type of activity, and the color describes the level of activity (see color bar). (EPS 21786 kb)

Supp Fig. 2. The conclusions do not depend on the size of the analysis window. The graph shows the activity of 133 hoverflies, separated into males (58) and females (75) as a function of the size of the analysis window, starting from 1 hour and moving up to 24 hours. The data for the 1-12 hour analysis windows come entirely from Day 2, starting from the middle of the day. The data for 13-24 hours include all of Day 2, as well as activity from Day 1. The graph shows that the mean activity decreases with the size of the analysis window, especially when the start and end of the day is included (from 11 hours and onwards), but the conclusions remain the same: i.e. there is no significant difference between male and female activity when using any of the analysis windows. This example confirms that the conclusions of the paper do not depend on the size of the analysis window. (EPS 223 kb)

Supp Fig. 3. Male and female hoverfly weights. The weight of 34 male and 23 female hoverflies. Even if the weights of the two sexes are not different from each other, note that the females have a larger spread towards larger sizes (75^th^ percentile for males 0.12 g vs 0.14 g for females, and the maximum at 0.14 g for males vs 0.18 g for females). (EPS 61 kb)

Supp Movie 1. The movie shows 8 LAMS tubes filmed from above, with 1 hoverfly in each tube. The data were manually scored, as shown in Supp Fig. 1. (WMV 30306 kb)
