# Supplementary figures and images for: Hoverfly locomotor activity is resilient to external influence and intrinsic factors
Source: J Comp Physiol A Neuroethol Sens Neural Behav Physiol. 2015 Nov 26;202:45–54. doi: 10.1007/s00359-015-1051-2 (PMC4698302; doi:10.1007/s00359-015-1051-2)

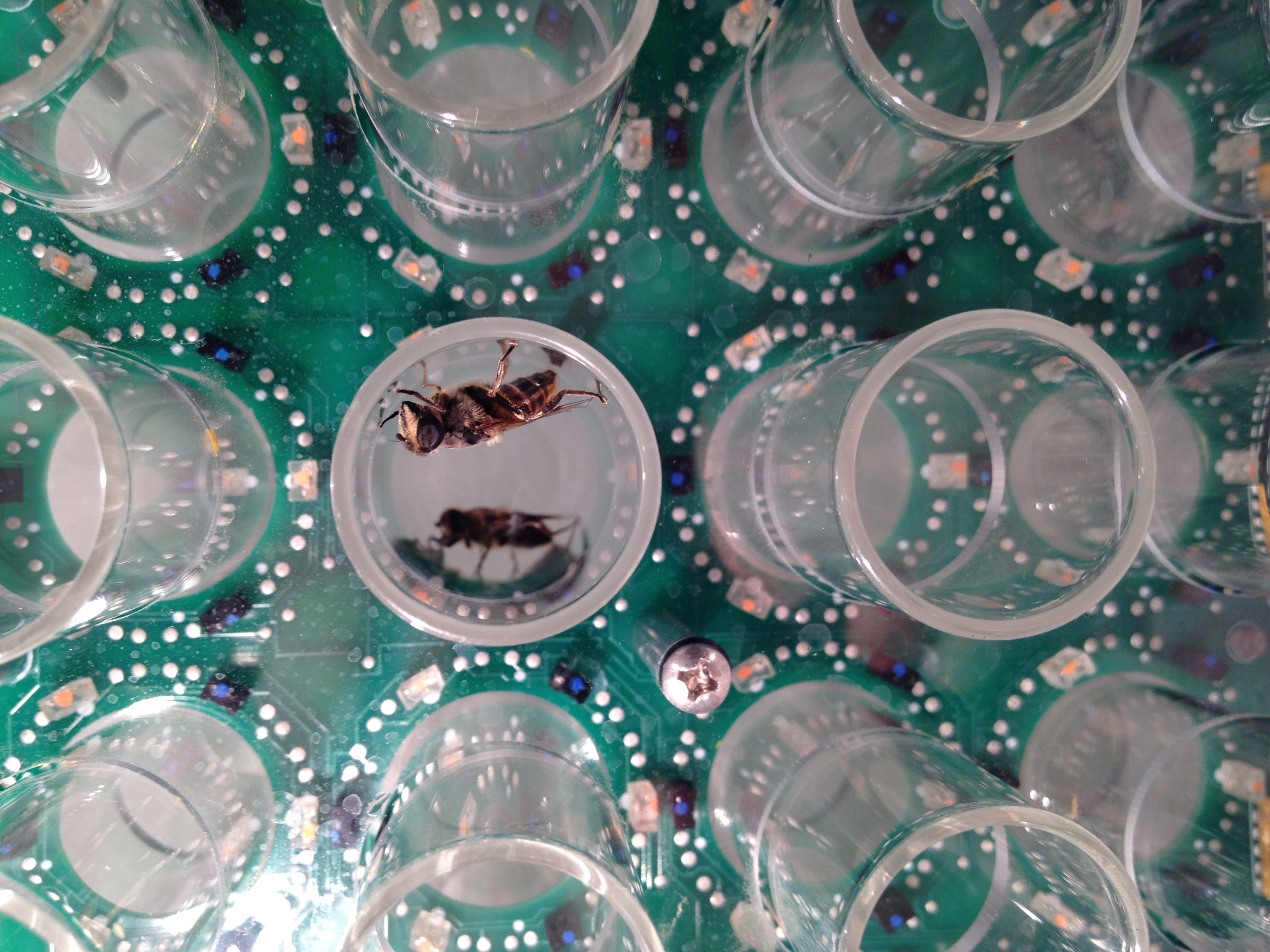

Supplement: Supplementary file 1 — Supplementary material 1 (JPEG 2331 kb) [file 359_2015_1051_MOESM1_ESM.jpg]
